# Supplementary material for: A new formulation of compartmental epidemic modelling for arbitrary distributions of incubation and removal times
Source: PLoS One. 2021 Feb 3;16(2):e0244107. doi: 10.1371/journal.pone.0244107 (PMC7857597; doi:10.1371/journal.pone.0244107)
Supplement: S1 Appendix — (PDF) [file pone.0244107.s001.pdf]

S1 Appendix: The uSEIR equations are delayed and the most efficient way to solve them numerically is to enlarge the number of functions at each time. We define $t_i^{\max} = n_i^{\max}\epsilon$ and $t_r^{\max} = n_r^{\max}\epsilon$ as the incubation and removal times, such that for $t_{i,r} > t_{i,r}^{\max}$ the distribution probabilities are negligible. These integers fix the number of additional variables we need to evolve at each time step: $E_1, ..., E_{n_i^{\max}}$ and $I_1, ..., I_{n_r^{\max}}$. The variables $E_k(t)$ and $I_k(t)$ measure the number of exposed of infected individuals at time t that will change compartment ($E \to I$ or $I \to R$) at time $t + k$.

The recursive relations for all these variables read:

- `E` compartments:

$$E_k(t+\epsilon) = \begin{cases} E_{k+1}(t) + \frac{r}{N} S(t) I(t)\, P_E(k\epsilon) & k = 1, \ldots, n_i^{\max} - 1 \\ \\ \frac{r}{N} S(t) I(t)\, P_E(t_i^{\max}) & k = n_i^{\max} \end{cases} \tag{48}$$

where $P_E(t)$ is the probability for t to be between t and $t + \epsilon$.

- `I` compartments:

$$I_k(t+\epsilon) = \begin{cases} I_{k+1}(t) + E_1(t)\, P_I(k\epsilon) & k = 1, \ldots, n_r^{\max} - 1 \\ \\ E_1(t)\, P_I(t_r^{\max}) & k = n_r^{\max} \end{cases} \tag{49}$$

While the SEIR variables:

$$E(t) = \sum_{k=1}^{n_i^{\max}} E_k(t), \quad I(t) = \sum_{k=1}^{n_r^{\max}} I_k(t). \tag{50}$$

and

$$\begin{aligned} S(t+\epsilon) &= S(t) - \frac{r}{N} S(t) I(t), \\ R(t+\epsilon) &= R(t) + I_1(t). \end{aligned} \tag{51}$$

Implementations of this algorithm in the Julia programming language and in Python/Cython can be found, respectively, in:

`https://gitlab.ift.uam-csic.es/alberto/useir`
`https://github.com/jjgomezcadenas/useirn`
