# Supplementary material for: A new formulation of compartmental epidemic modelling for arbitrary distributions of incubation and removal times
Source: PLoS One. 2021 Feb 3;16(2):e0244107. doi: 10.1371/journal.pone.0244107 (PMC7857597; doi:10.1371/journal.pone.0244107)
Supplement: Attachment — Submitted filename: AnswerToReviewers.docx [file pone.0244107.s002.docx]

Academic Editor Decision:

**Although Reviewer 1 has given favourable comments, Reviewer 2 has raised many valid concerns that I happen to fully agree with.**

Reviewers

**Comments to the Author**

**1. Is the manuscript technically sound, and do the data support the conclusions?

The manuscript must describe a technically sound piece of scientific research with data that supports the conclusions. Experiments must have been conducted rigorously, with appropriate controls, replication, and sample sizes. The conclusions must be drawn appropriately based on the data presented.**

**Reviewer #1: Yes**

**Reviewer #2: Yes**

**2. Has the statistical analysis been performed appropriately and rigorously?**

**Reviewer #1: I Don't Know**

**Reviewer #2: Yes**

**3. Have the authors made all data underlying the findings in their manuscript fully available?

The** [**PLOS Data policy**](http://www.plosone.org/static/policies.action#sharing) **requires authors to make all data underlying the findings described in their manuscript fully available without restriction, with rare exception (please refer to the Data Availability Statement in the manuscript PDF file). The data should be provided as part of the manuscript or its supporting information, or deposited to a public repository. For example, in addition to summary statistics, the data points behind means, medians and variance measures should be available. If there are restrictions on publicly sharing data—e.g. participant privacy or use of data from a third party—those must be specified.**

**Reviewer #1: Yes**

**Reviewer #2: Yes**

**4. Is the manuscript presented in an intelligible fashion and written in standard English?

PLOS ONE does not copyedit accepted manuscripts, so the language in submitted articles must be clear, correct, and unambiguous. Any typographical or grammatical errors should be corrected at revision, so please note any specific errors here.**

**Reviewer #1: Yes**

**Reviewer #2: Yes**

**5. Review Comments to the Author**

**Reviewer #1: This is an interesting and I think quite useful approach to a long-lamented issue in compartmental models for infectious diseases, that of the non-exponentially distributed transition times. I appreciate the work that has gone into the approach.**

**My only large comment is on the use of the word uniform. I originally misinterpreted that to mean uniformly distributed, when I believe you meant homogenous or fixed. I would recommend changing the wording.**

ANSWER: We have changed the wording as requested (changes in red):

Page 2: uniformity of -> fixed values of

Page 7: uniform limit -> limit of fixed values of the parameters

non-uniformities -> non-homogeneities

Non-uniform -> Generically distributed
 Non-uniformities-> Non-trivial distributions for ti and tr

**Specific comments**:

**Pg 1: SEIR is susceptible-exposed-infectious-removed**

ANSWER: We have corrected it.

**Pg 3: It was not clear whether this ABM used fixed values for t_i or t_r**

ANSWER: We performed several simulations, some with fixed t_i and t_r (shown in Fig.3 ) and also assuming various distributions (Fig.4 exponential distributions, Fig. 6 gamma distribution and Fig. 7 negative binomial)

**Pg 16: The last line appears to be missing some text**

ANSWER: Yes, this is a typo. We have corrected it.

**Reviewer #2: The authors present a formulation of compartmental modelling to include arbitrary distributions of incubation and removal times. While the authors claim that the conventional paradigm for compartmental epidemiological modelling is the SEIR model, and that this model assumes exponentially distributed incubation and removal times.** **A minor point: SEIR is not the conventional paradigm for compartmental epidemiological modelling. It is one type of compartmental model structure, but its appropriateness depends on 1) the pathogen being studied, 2) the research question, and 3) data availability. There are many different model structures (SI, SIS, SIR, SEIR) or adaptions thereof (SIIBS, SEIRS, SEIHFR), and individuals can have different states than just S, E, I, and R, although it is recognized to be an appropriate model choice for Covid-19.**

ANSWER: We have corrected the abstract accordingly.

**Nonetheless, the assumption that incubation and other transition times are exponentially distributed is indeed well known. However, there already exist various types of distribution delay models and renewal equations, which can cover all alternatively assumed distributions, in a similar fashion as the authors propose in this paper.**

ANSWER: We do not understand what the criticism is here, since we clearly state in the introduction:

“It is well-known that these more general distributions are difficult to be captured by classical compartmental models such as SEIR, which normally treat the incubation and removal times as exponentially distributed, or as the sum of several exponentially-distributed independent times. “

This is the starting point, not what we do ! We present a formulation that is different to what can be found in the previous literature.

**Even the 1927 Kermack and McKendrick model allows for this. They generally all require the introduction of additional strata in the model, the capability to track the time at which individuals were infected, and some sort of integro-differential equation to solve them (see e.g. Breda et al 2012 On the formulation of epidemic models (an appraisal of Kermack and Mckendrick)). Although they are more realistic, this comes at the added cost of computational inefficiency, which can be a real bottleneck when hundreds of thousands of models need be ran to e.g. fit the model to observed data.**

ANSWER: This is a criticism to previous work on the subject. Our paper presents a solution to the compartmental epidemic modelling for arbitrary distributions of incubation and removal times that is different to any approach we have seen in the literature, and we have demonstrated that it  is numerically efficient, providing verifiable evidence of this: our software code can be freely downloaded and tested.

A run of 10^5 models takes a few CPU*hours, without any particular optimization, and we are presently using it for fitting real data without any problem. We invite the reviewer to check our algorithm and code for him/herself, since we provide all what is needed.

**It is this reason why many modellers opt for a sequence of e.g. different infectious compartments to implement an Erlang rather than exponential distribution, as a better trade-off between realism and computational efficiency. Although this is not the case for the initially presented uSEIR model with homogeneous values for ti and tr for all individuals in the population, this model formulation is probably even more unrealistic than the assumption regarding exponential distributions, so there would always be a need to incorporate multiple strata in the model.**

ANSWER: The software that we present is for the general case, not just for uSEIR, and as explained above, it is highly efficient.

**There are several other, smaller points:**
**- The authors refer to agents in their ABM model as 'turtles' to stick with the nomenclature of this specific software package, which is fine, but it would be good to stick to other more basic epidemiological notation as well to improve readability of the manuscript for individuals with an epidemiological background, which I assume is the target audience for this paper. E.g., the effective contact rate (which multiplied with I(t) is the force of infection) is referred to as the infection rate, and denoted as "Rs->e" rather than the more conventional symbols using c, Beta or Lambda. When a susceptible individual is infected, the authors refer to this as being exposed (the Exposed compartment in the SEIR is an unfortunate standard naming convention, and is better referred to as Preinfectious).**

ANSWER: The first time it is mentioned, we have added exposed/preinfectious on page 1. We have added a footnote on page 6 to indicate the relation of rS-> E to the SEIR parameter beta.

**- The authors find "an interesting observation is that most of the observed variance of the outbreaks is a simple time translation". This is a well known phenomenon as the stochastic effects of an epidemic are especially important when modelling 1) an early outbreak or 2) a small population. Related, on page 14, authors have observed that, with a given R0, the peak and shape of the epidemic does not differ substantially. For immunizing infections, it is indeed a well known fact, as the epidemic will peak once the herd immunity threshold is reached, or stated otherwise, when the proportion of the population that is susceptible is equal to 1 /R0.**

ANSWER: The point that we make is that the time translation is not something that the solution of the SEIR equations captures, so when the non-universal time shift is corrected, the agreement between the average of simulated epidemic evolution and the solution to SEIR is much better. We furthermore have found an analytic asymptotic solution that represents the shape of the curve extremely well. We have not seen this in the literature. If it is, we would appreciate if the reviewer would point to the references.

**- The usual terminology for the universal equation starting at page 14 is the 'final size equation'. On page 12, the authors observe that the total number of infections exceed the herd-immunity threshold. This is another well known phenomena and is usually referred to as an epidemic 'overshooting'.**

ANSWER: we have made the following change on page 13

This is because-> The reason for this well known overshooting is that

**- What does theta in equation 9 refer to?**

ANSWER: Theta(t) is the Heaviside function. We have added after the equation on page 7.

**- Authors give estimates for R0 for Covid-19 of 3 and 3.5. As R0 values are extremely context specific, it would be good to state in which populations these values have been estimated.**

ANSWER: Nowhere in the paper have we claimed to be doing a realistic description of COVID-19 epidemic. We illustrate our results with a value of R0 which was similar to the numbers that were being quoted for COVID in the first wave in several countries, but the specific choice is not relevant for the results of our paper. Of course, we are well aware that R0 depends on many factors.

To avoid confusion we have rephrased slightly the paragraph on page 13, where we refer to R0.

**- On page 12, authors refer to "individuals for which the probability of infection is zero, which in practice makes them immune". They are here referring to infected individuals, which by definition means they are not immune, so I would at least use a different term for these individuals. Related to this, the authors claim that due to the estimated negative binomial distribution of R0, 60% of the population must be immune. I see how the 60% value is calculated from the CDF, but do not understand this interpretation. Yes, many individuals will have a low number of contacts and will therefore only infect few (or some 0) secondary cases, but this does not mean that they are immune, which would imply they would not have been infected themselves. In fact, the ones resulting in few secondary cases are probably the very young or very old, which are the age-groups at highest risk for severe disease and death, so they would leave a very real mark on the epidemic progression once other outcomes than just infections are incorporated.**

ANSWER: We agree that the name immune could be confusing. We have modified this reference at the top of page 13.
