# Supplementary material for: A new formulation of compartmental epidemic modelling for arbitrary distributions of incubation and removal times
Source: PLoS One. 2021 Feb 3;16(2):e0244107. doi: 10.1371/journal.pone.0244107 (PMC7857597; doi:10.1371/journal.pone.0244107)
Supplement: Attachment — Submitted filename: Answer_to_referee_2.docx [file pone.0244107.s003.docx]

**While the work meets the criteria of PLOS ONE, you should provide an improved introduction that places your work into historical context. Reviewer 2 provided a guide for this context by pointing out the generalizability of Kermack and McKendrick's work as well as the associated difficulties. Also, your notation uSEIR seems arbitrary - does the "u" stand for something. If so, please describe. If not, it would be worthwhile to use meaningful nomenclature.**

ANSWER: Following the suggestion of Referee 2, we have modified a paragraph in the introduction, referring explicitly to Kermack-McKendrick seminal work. We have also explained that the “u” in uSEIR, refers to unitary, because our formulation makes it explicit that the inter-compartment transition rates conserve the total probability as time evolves. In order to simplify the reading we have taken out the reference to uSEIR in the abstract.
